# The *in vitro* antiviral activity of lactoferrin against common human coronaviruses and SARS-CoV-2 is mediated by targeting the heparan sulfate co-receptor

**DOI:** 10.1080/22221751.2021.1888660

**Published:** 2021-02-27

**Authors:** Yanmei Hu, Xiangzhi Meng, Fushun Zhang, Yan Xiang, Jun Wang

**Affiliations:** a Department of Pharmacology and Toxicology, College of Pharmacy, The University of Arizona, Tucson, AZ, USA; b Department of Microbiology, Immunology and Molecular Genetics, University of Texas Health Science Center at San Antonio, San Antonio, TX, USA

**Keywords:** SARS-CoV-2, COVID-19, lactoferrin, heparan sulfate, heparin

## Abstract

Coronavirus disease 2019 (COVID-19) is an ongoing pandemic that lacks effective therapeutic interventions. SARS-CoV-2 infects ACE2-expressing cells and gains cell entry through either direct plasma membrane fusion or endocytosis. Recent studies have shown that in addition to ACE2, heparan sulfate proteoglycans (HSPGs) also play an important role in SARS-CoV-2 cell attachment by serving as an attachment factor. Binding of viral spike protein to HSPGs leads to the enrichment of local concentration for the subsequent specific binding with ACE2. We therefore hypothesize that blocking the interactions between viral spike protein and the HSPGs will lead to inhibition of viral replication. In this study, we report our findings of the broad-spectrum antiviral activity and the mechanism of action of lactoferrin (LF) against multiple common human coronaviruses as well as SARS-CoV-2. Our study has shown that LF has broad-spectrum antiviral activity against SARS-CoV-2, HCoV-OC43, HCoV-NL63, and HCoV-229E in cell culture, and bovine lactoferrin (BLF) is more potent than human lactoferrin. Mechanistic studies revealed that BLF binds to HSPGs, thereby blocking viral attachment to the host cell. The antiviral activity of BLF can be antagonized by the HSPG mimetic heparin. Combination therapy experiment showed that the antiviral activity of LF is synergistic with remdesivir in cell culture. Molecular modelling suggests that the N-terminal positively charged region in BLF (residues 17-41) confers the binding to HSPGs. Overall, LF appears to be a promising drug candidate for COVID-19 that warrants further investigation.

## Introduction

The coronavirus disease 2019 (COVID-19) has led to more than 100 million infections and over 2.1 million deaths worldwide, and more than 25 million infections and over 429,000 deaths in the US alone as of 28 January, 2021, rendering it one of the most life-threatening infectious disease outbreaks in human history. Severe acute respiratory syndrome coronavirus 2 (SARS-CoV-2), the causative agent of COVID-19 [1], together with severe acute respiratory syndrome coronavirus (SARS-CoV) [2], and Middle East respiratory syndrome coronavirus (MERS-CoV) [3], are the three highly pathogenic human coronaviruses that cause severe respiratory syndrome, while the other four common human coronaviruses (HCoV-229E, HCoV-NL63, HCoV-OC43 and HCoV-HKU1) account for 15–30% global cases of common cold in humans [4]. Limited number of therapeutics including vaccines and small molecules are available for COVID-19 treatment. As future coronavirus outbreak is highly possible, it is desired to develop broad-spectrum antivirals that are suitable for the prevention and treatment of both current circulating CoVs and future emerging CoVs.

Lactoferrin (LF), a naturally occurring, non-toxic iron-binding glycoprotein present in several mucosal secretions, plays an important role in the first line of defence against microbial infections [5]. It is known that LF has broad-spectrum antiviral activity against a wide range of human and animal viruses including both DNA and RNA viruses [6,7]. Moreover, the anti-inflammatory and immunomodulatory activities of LF may have additional benefits in severe infections [8]. Furthermore, the presence of an intestinal receptor for the uptake of LF following oral administration [9], resistance to proteolytic degradation by trypsin and trypsin-like enzymes [10], and several established oral delivery systems for LF [11], ensures its oral bioavailability.

The reported antiviral mechanisms of LF include (1) direct binding to viral protein and inhibition of the adsorption of virus to the target cells [12–14]; (2) binding to heparan sulfate proteoglycans (HSPGs) on the host cell surface, which reduces viral attachment and subsequent viral entry [15–19]. Heparan sulfate (HS) is a linear and sulfated polysaccharide that is abundantly expressed on the surface of almost all cell types in the forms of HSPGs. The negatively charged HSPGs often serve as an attachment factor for a diverse of viruses [20,21]; (3) interfere with intracellular trafficking of virus [22]. LF was reported to have antiviral activity against SARS-CoV [17]. However, the broad-spectrum antiviral activity and antiviral mechanism of action of LF against common human coronaviruses as well as SARS-CoV-2 have not been systematically studied and therefore warrants further investigation. In this work, we profiled the broad-spectrum antiviral activity of LF against multiple common human coronaviruses including HCoV-OC43, HCoV-NL63, and HCoV-229E as well as SARS-CoV-2 and its mechanism of action. It was found that LF inhibits not only SARS-CoV-2, but also HCoV-OC43, HCoV-NL63, and HCoV-229E. The antiviral mechanism of action of LF was found to be mediated through binding to HSPGs on the host cell surface, thereby preventing viral attachment to the host cells. Several recent studies suggest that HSPGs serve as an attachment factor for the initial tethering of SARS-CoV-2 spike protein to host cell membrane and facilitates the subsequent binding to the specific receptor ACE2 [23–26]. Specifically, drug time-of-addition experiment and SARS-CoV-2 pseudovirus assays indicated that LF exerts its antiviral activity by blocking viral attachment to target cells. In addition, LF has direct binding to heparin, a mimetic of HSPGs, and pre-mixing LF with heparin decreased the inhibitory activity of LF on viral attachment and reduced antiviral activity of LF in cell culture. Furthermore, we have shown that LF has synergistic antiviral effect with remdesivir, which further warrants its development as a potential anti-coronavirus agent against both current circulating and future emerging coronaviruses.

## Results

### Both bovine and human lactoferrins have broad-spectrum antiviral activity against multiple HCoVs in cell culture

**Figure 1.**
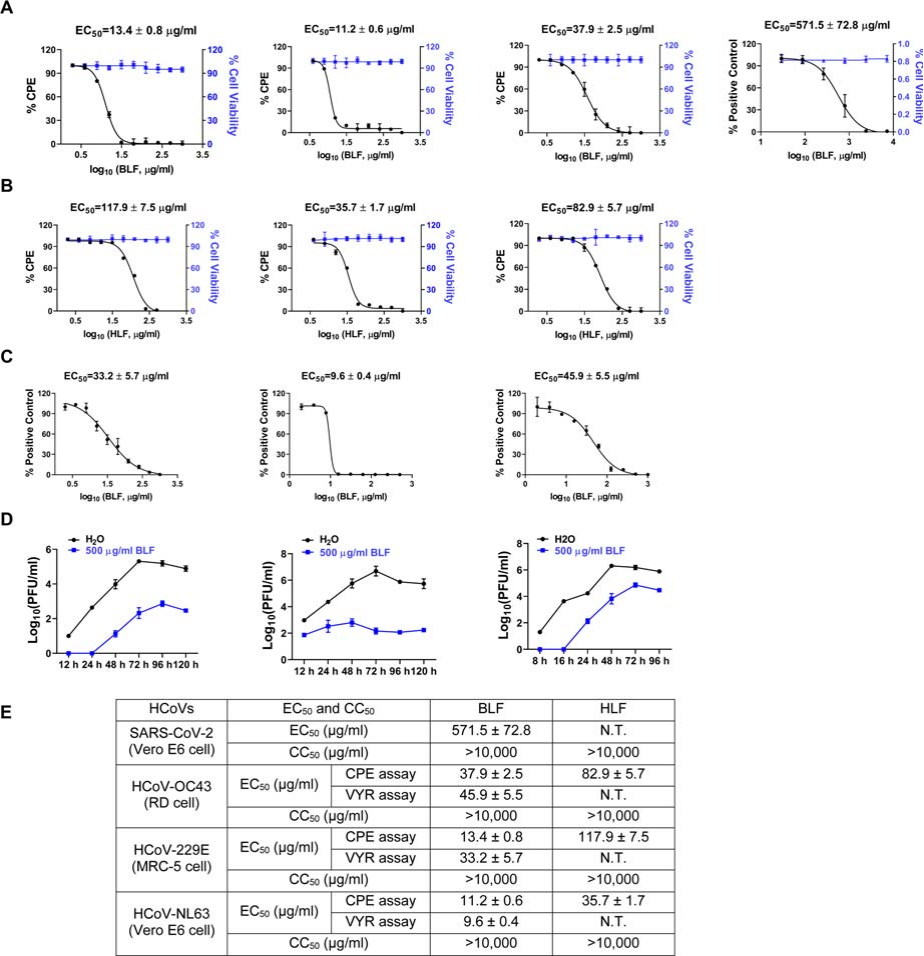
Antiviral activity of bovine and human lactoferrins against multiple common HCoVs and SARS-CoV-2 in cell culture. (A) The antiviral activity of bovine lactoferrin (BLF) against HCoV-229E, HCoV-NL63, HCoV-OC43 in CPE assay and SARS-CoV-2 in immunofluorescence imaging assay (from left to right). (B) The antiviral activity of human lactoferrin (HLF) against HCoV-229E, HCoV-NL63, HCoV-OC43 in CPE assay (from left to right). (C) Secondary viral yield reduction (VYR) assay of BLF against HCoV-229E, HCoV-NL63, HCoV-OC43 (from left to right). (D) Growth curves of HCoV-229E, HCoV-NL63, HCoV-OC43 (from left to right) in the absence (black) or presence of 500 µg/ml BLF (blue). EC_50_ curve fittings in the CPE and VYR assays were obtained using log_10_ (concentration of inhibitors) vs. percentage of CPE or percentage of positive control with variable slopes in prism 8. The cellular cytotoxicity test was included in CPE experiment and the resulting curves were shown in blue. All data are mean ± standard deviation of three replicates. (E) EC_50_ and CC_50_ values of BLF and HLF against multiple HCoVs and SARS-CoV-2 in cell culture.

The antiviral activity of bovine and human lactoferrins was first tested in cytopathogenic effect (CPE) assay in cell culture against multiple HCoVs, including HCoV-229E, HCoV-NL63, and HCoV-OC43. Bovine lactoferrin (BLF) exhibited potent antiviral activity against all three HCoVs tested, with 50% effective concentration (EC_50_) values ranging from 11.2 to 37.9 µg/ml (Figure 1(A,E)). Human lactoferrin (HLF) also showed potent antiviral activity against all three HCoVs tested, however, it was about 3–8 folds less potent than BLF with EC_50_ values ranging from 35.7 to 117.9 µg/ml (Figure 1(B,E)). Both BLF and HLF were not cytotoxic to the cells at the concentration ranges tested (Figure 1(A–D), blue lines). Significantly, BLF also inhibited SARS-CoV-2 replication in Vero E6 cells with an EC_50_ value of 571.5 ± 72.8 µg/ml in the immunofluorescence imaging assay (Figure 1(A,E)). Given the higher inhibitory potency of BLF versus HLF, BLF was chosen for the following experiments. To confirm the antiviral activity of BLF, a secondary viral yield reduction (VYR) assay was performed to determine the effect of BLF treatment on infectious virus production. As measured in plaque assay, BLF dose-dependently inhibited infectious virion production of HCoV-229E, HCoV-NL63, and HCoV-OC43 in cell culture at 2 days post infection (dpi), with EC_50_ values ranging from 9.6 to 45.9 µg/ml (Figure 1(C,E)). Next, to test the inhibitory effect of BLF on viral replication over time, HCoV-229E, HCoV-NL63, and HCoV-OC43 were amplified with and without BLF in MRC-5, Vero E6, and rhabdomyosarcoma (RD) cells, respectively, and the viral titers in the cell culture supernatants were quantified at different time points post-viral infection by plaque assay (Figure 1(D)). It was found that BLF decreased the viral titers of all three viruses by more than 2 log_10_ units at all time points, and it significantly inhibited the viral replication of HCoV-NL63, as there was no obvious viral titer increase up to 120 h post infection (hpi) (Figure 1(D), middle panel). Taken together, both BLF and HLF had potent antiviral activity against multiple common HCoVs and SARS-CoV-2, indicating LF is a promising antiviral drug candidate.

### BLF inhibits infectious HCoV-OC43 and SARS-CoV-2 pseudovirus replication by blocking viral attachment to the host cell

**Figure 2.**
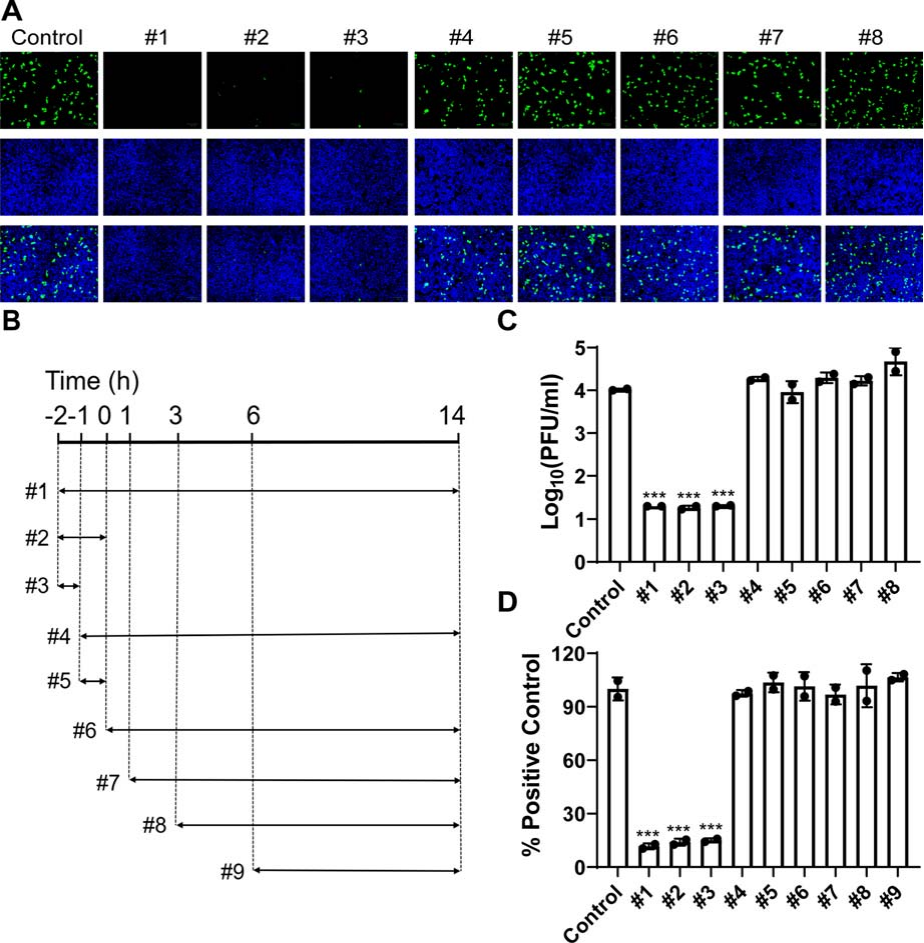
Time-of-addition experiments of BLF with HCoV-OC43 or SARS-CoV-2 pseudovirus particles. (A) Representative images of intracellular HCoV-OC43 virus detected by immunofluorescence staining using HCoV-OC43 specific antibody. Images were taken by Zoe^TM^ Fluorescent Cell Imager (BioRad). (B) Illustration of the time periods when BLF (1000 µg/ml) was present in the time-of-addition experiments. Arrows represent the periods of time that BLF was present in the cell culture. (C) Quantification of HCoV-OC43 virus released into the cell culture medium using plaque assay. (D) Relative SARS-CoV-2 pseudovirus particle titers were quantified by measuring luciferase activity using Bright-Glo Luciferase Assay System and normalized to control. ***, *p* < 0.001 (student's *t*-test). Data are mean ± standard deviation of two replicates.

To elucidate the antiviral mechanism of action of BLF, the drug time-of-addition experiments were carried out to determine at which step(s) of viral replication BLF exerts its antiviral activity (Figure 2(A–D)). For this, 1000 µg/ml of BLF was added to the cell culture at different time points of viral replication (Figure 2(B)) including viral attachment and onwards (#1), viral attachment and entry (#2), viral attachment only (#3), viral entry and onwards (#4), viral entry only (#5), and different time points post-viral entry (#6–#9). For this mechanistic study, HCoV-OC43 was chosen as a representative example of common human coronaviruses, and SARS-CoV-2 pseudovirus was used as a surrogate for the infectious SARS-CoV-2. In the first set of time-of-addition experiment, RD cells were infected with HCoV-OC43 at an MOI of 1, and intracellular viral protein expression was quantified by immunofluorescence staining using HCoV-OC43 specific antibody (Figure 2(A)), and viral titers of progeny virus released into the cell culture medium were quantified by plaque assay (Figure 2(C)). The immunofluorescence assay results showed that BLF only inhibited viral replication when it was included in the viral attachment stage (Figure 2(A), #1, #2, and #3), and it had no significant antiviral effect when added during the viral entry and post-viral entry (Figure 2(A), #4–#9). Consistent with the immunofluorescence assay results, viral titers were significantly decreased when BLF was present at steps including viral attachment (Figure 2(C), #1-#3), but not in the entry and post-entry steps (Figure 2(C), #4–#8). To test whether BLF inhibits SARS-CoV-2 through a similar mechanism, SARS-CoV-2 pseudovirus particles were used in the second set of time-of-addition experiment. The relative titers of SARS-CoV-2 pseudoviral particles were determined by measuring the ratio of luciferase reporter gene expression level with and without LF treatment. The results demonstrated that BLF inhibited SARS-CoV-2 pseudovirus replication at the attachment stage (Figure 2(D), #1–#3), but not viral entry and post-entry stages (Figure 2(D), #4–#9). Collectively, the drug time-of-addition results suggested that LF blocks viral attachment to host cells and has no effect on viral replication when added post-viral entry.

### LF inhibits SARS-CoV-2 pseudovirus replication in multiple cell lines

**Figure 3.**
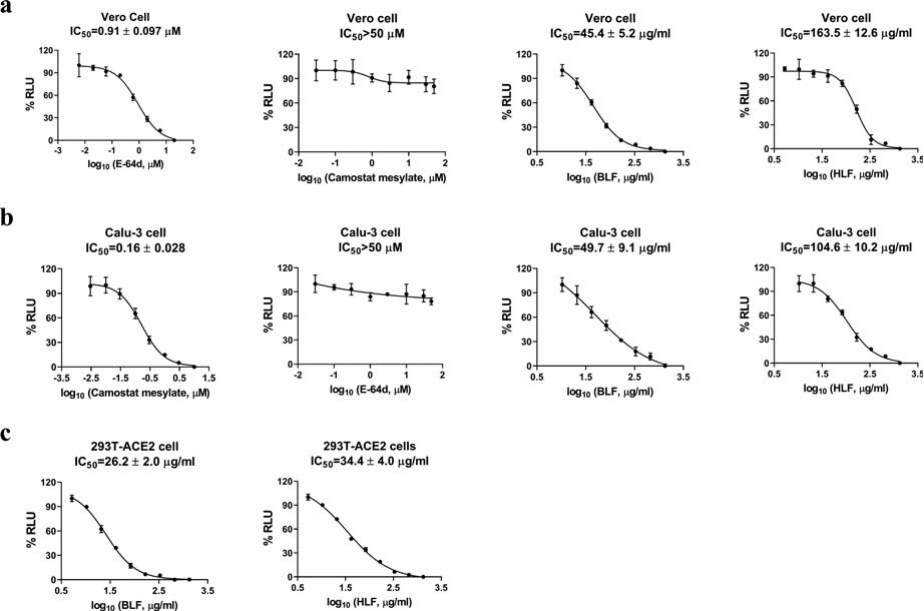
Inhibitory activity of BLF and HLF in the SARS-CoV-2 pseudovirus neutralization assay in different cell lines. (A) Vero E6 cell. (B) Calu-3 cell. (C) 293T cell overexpressing ACE2. Cathepsin L inhibitor E-64d and TMPRSS2 inhibitor camostat mesylate were included as controls in Vero E6 and Calu-3 cells, respectively. IC_50_ curve fittings using log_10_ (concentration of inhibitors) vs. percentage of DMSO control with variable slopes were performed in Prism 8. All data are mean ± standard deviation of two replicates.

Pseudovirus neutralization assay is an established model to study the mechanism of viral entry into host cells and has been widely used to assess the antiviral activity of viral entry/fusion inhibitors [27–29]. To test whether the antiviral effect of lactoferrins against SARS-CoV-2 is cell type dependent, BLF and HLF were tested in SARS-CoV-2 pseudovirus assay in three different types of cell lines: Vero E6 cell (Figure 3(A)), Calu-3 cell (Figure 3(B)), and 293T cell overexpressing ACE2 (293T-ACE2) (Figure 3(C)). Vero E6 and 293T-ACE2 cells express high levels of ACE2 on the apical membrane domain but minimal levels of transmembrane serine proteinase 2 (TMPRSS2), the host serine protease that cleaves viral spike protein [30]. As such, SARS-CoV-2 virus enters these cells through endocytosis and relies on endosomal cathepsin L for viral spike protein activation [31,32]. In contrast, Calu-3 is a human lung epithelial cell line with endogenous expression of both ACE2 and TMPRSS2 [33], and SARS-CoV-2 spike protein can be activated at the cell surface by TMPRSS2, resulting in direct cell entry at the plasma cell membrane. Previously reported cathepsin L inhibitor E-64d and TMPRSS2 inhibitor camostat mesylate were included as controls for the SARS-CoV-2 pseudovirus entry assays. It was found that both BLF and HLF inhibited SARS-CoV-2 pseudovirus entry in a dose-dependent manner in all three cell lines, with IC_50_ values ranging from 26.2 to 49.7 µg/ml and 34.4–163.5 µg/ml, respectively (Figure 3). BLF was more potent than HLF, which agrees with the antiviral assay results from the infectious HCoVs (Figure 1). These results indicate that the inhibition of SARS-CoV-2 pseudovirus entry by lactoferrins is cell type independent.

### LF blocks viral attachment through interaction with the host cell instead of the virus

**Figure 4.**
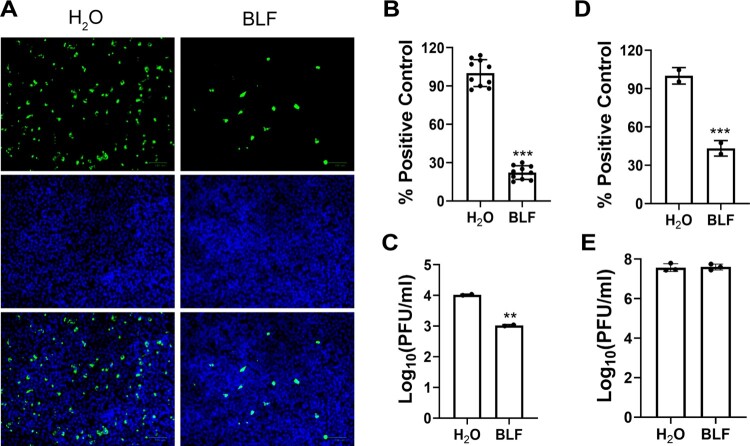
Evaluation of the direct effect of BLF on host cells or viral particles through pretreatment of cells or virus. (A) Representative immunofluorescence images of pretreating cells with 1000 µg/ml BLF or H_2_O. (B) Quantification of the results from panel (A). (C) Viral titers of HCoV-OC43 from cell culture medium of RD cells pre-treated with H_2_O or 1000 µg/ml BLF. (D) Expression levels of luciferase reporter gene in SARS-CoV-2 pseudovirus particles infected Vero E6 cells pre-treated with H_2_O or 1000 µg/ml BLF. (E) Infectious viral titers of HCoV-OC43 virus pre-treated with 1000 µg/ml BLF or H_2_O. **, *p* < 0.01; ***, *p* < 0.001 (student's *t*-test). Data are mean ± standard deviation of three replicates.

SARS-CoV-2 attaches to the host cells through the interactions between viral spike protein and host cell ACE2 receptor and HSPGs attachment factor [23]. To determine whether LF interferes with viral attachment through interacting with the host cell or the virus, we used the HCoV-OC43 virus or the SARS-CoV-2 pseudovirus particles as SARS-CoV-2 surrogates and performed cell pretreatment and virucidal experiments. The results demonstrated that pretreatment of RD cells with 1000 µg/ml BLF prior to viral infection reduced intracellular viral protein expression level by about 80% compared to the H_2_O-treated control sample (Figure 4(A,B)), and the viral titer in the supernatant was decreased by about 1 log_10_ unit in the presence of 1000 µg/ml BLF (Figure 4(C)). Similarly, pretreatment of Vero E6 cells with 1000 µg/ml BLF decreased the SARS-CoV-2 pseudovirus luciferase activity to about 50% of the H_2_O-treated control (Figure 4(D)). To assess the direct effect of LF on HCoV-OC43 viral particles, HCoV-OC43 viruses were pre-treated with 1000 µg/ml BLF or same volume of sterile H_2_O (untreated control) at 37 °C for 3 h, followed by viral titer quantification by plaque assay in RD cells. It was found that BLF-treated virus showed the same number of plaques as the H_2_O-treated control at 10^−6^-fold dilution (Figure 4(E)). The final concentration of BLF in the plaque assay was 0.001 µg/ml, far below its minimum inhibitory concentration (EC_50_ = 37.9 ± 2.5 µg/ml) and thus had no effect on plaque formation. Taken together, these results suggested that BLF inhibits viral attachment through binding to host cells instead of the virus.

### BLF and HLF bind to heparin *in vitro*

**Figure 5.**
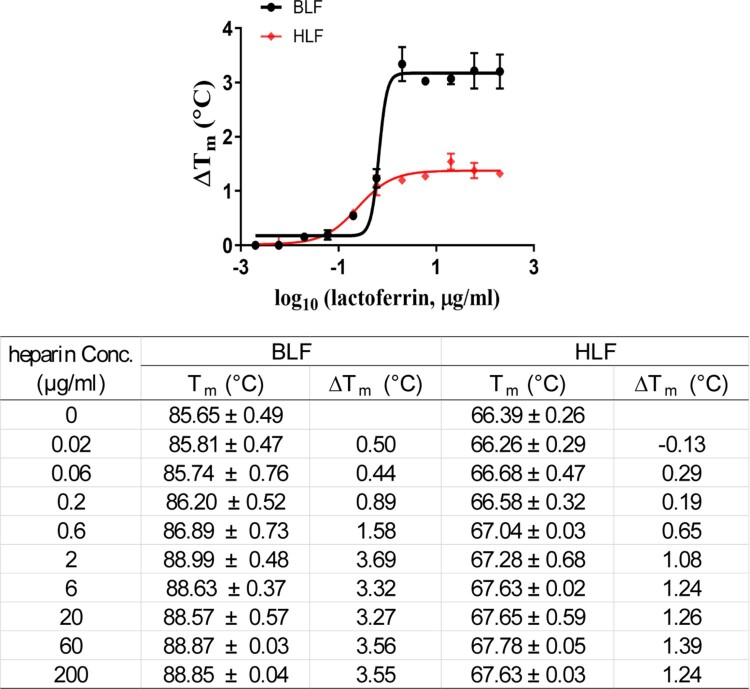
Effect of heparin on melting temperature (*T_m_*) of BLF and HLF. Data were plotted with Δ*T_m_* vs. log_10_ (concentrations of heparin) using Boltzmann Sigmoidal equation in Prism 8 (left). *T_m_* of lactoferrins in the absence or presence of indicated concentrations of heparin are shown in the table, and *ΔT_m_*s were calculated by subtracting the *T_m_* of LF without heparin. Data are mean ± standard deviation of two replicates.

Previous study reported that LF blocks SARS-CoV pseudovirus infection in HEK293E/ACE2-Myc cells by binding to HSPGs on the cell surface [17]. In addition, HCoV-NL63 was shown to utilize HSPGs as adhesion receptor for viral attachment to target cells through its interaction with the membrane (M) protein [18,19]. Recently, cell surface HSPGs were discovered as the co-receptors for SARS-CoV-2 spike (S) protein and facilitate the subsequent binding to ACE2 receptor [23,24,26]. Based on these findings and our results listed above, we hypothesize that LF exerts its broad-spectrum antiviral activity against coronaviruses by binding to HSPGs, therefore indirectly blocking the interaction between viral spike protein and ACE2. To test this hypothesis, we chose heparin (Sigma Cat.# H3393) as a mimetic of HSPGs, and performed the differential scanning fluorimetry (DSF) assay [34] to determine the direct binding of heparin to BLF and HLF. Specific binding of a ligand to a protein typically stabilizes the target protein, resulting in an increased melting temperature (*T*_m_). DSF results demonstrated that heparin increased the *T*_m_ of both BLF and HLF in a dose-dependent manner (Figure 5), indicating the direct binding of BLF and HLF to heparin. In addition, BLF displayed higher binding affinity than HLF to heparin as shown by the larger ΔT_m_, and this result agrees with the more potent antiviral activity of BLF compared to HLF.

**Figure 6.**
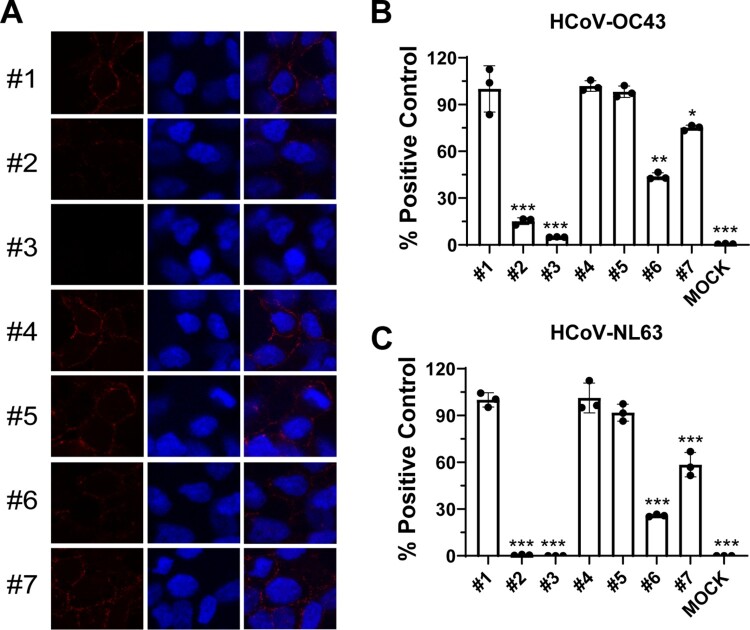
Heparin reduced the inhibitory activity of BLF on viral attachment to target cells. (A) Representative immunofluorescence images of HCoV-OC43 attached to RD cell surface detected by immunofluorescence staining. (B) Quantification of HCoV-OC43 attached to RD cell surface detected by RT-qPCR of N gene. (C) Quantification of HCoV-NL63 attached to Vero E6 cell surface detected by RT-qPCR of N gene. #1: H_2_O; #2: 500 µg/ml BLF; #3: 1000 µg/ml BLF; #4: 10 µg/ml heparin; #5: 30 µg/ml heparin; #6: 500 µg/ml BLF+10 µg/ml heparin; #7: 500 µg/ml BLF+30 µg/ml heparin. *, *p* < 0.05; **, *p* < 0.01; ***, *p* < 0.001 (student's *t*-test). All data are mean ± standard deviation of three replicates.

Next, to confirm that BLF blocks viral attachment to target cells, a viral attachment assay was carried out in the presence of different combinations of LF and/or heparin, and the attached HCoV-OC43 or HCoV-NL63 on the surface of RD cells or Vero E6 cells were quantified by immunofluorescence staining (Figure 6(A)) and real-time PCR (Figure 6(B,C)). Fluorescent signals were detected on the surface of RD cells that were treated with H_2_O control (Figure 6(A), #1), indicating the binding of HCoV-OC43 virus to the host cell surface. BLF-treated samples showed dose-dependent decrease of fluorescent signals on the cell surface (Figure 6(A), #2–#3), suggesting BLF inhibited viral attachment. Heparin treatment alone had no significant effect on viral attachment (Figure 6(A), #4–#5) as shown by the immunofluorescence intensity. In contrast, pretreatment of BLF with heparin before adding the mixture to the viral attachment assay abolished the inhibition of viral attachment as the fluorescence signals were restored (19% at 10 µg/ml and 86% at 30 µg/ml of heparin) (Figure 6(A), #6–#7). No specific antibody against HCoV-NL63 was available, so the immunofluorescence assay was not performed for HCoV-NL63. Instead, we quantified the amount of cell surface-attached viruses by RT-qPCR. Both HCoV-OC43 and HCoV-NL63 viral RNA levels were significantly reduced in a dose-dependent manner with BLF treatment alone: ∼15% and ∼5% of HCoV-OC43 viral RNA were detected with 500 and 1000 µg/ml BLF treatment; ∼0.5% and ∼0% of HCoV-NL63 viral RNA were detected with 500 and 1000 µg/ml BLF treatment (Figure 6(B,C)). Heparin itself had no obvious effect on viral RNA levels at the indicated concentrations (10 and 30 µg/ml) (Figure 6(B,C)). However, BLF lost partial potency in the presence of 10 or 30 µg/ml heparin: ∼45% or ∼75% of HCoV-OC43 viral RNA and ∼25% or 60% HCoV-NL63 viral RNA were detected under these conditions (Figure 6(B,C)). Collectively, the results suggest that BLF interferes viral attachment through its interaction with cell surface HSPGs.

### Heparin decreases antiviral activity of BLF in cell culture

**Figure 7.**
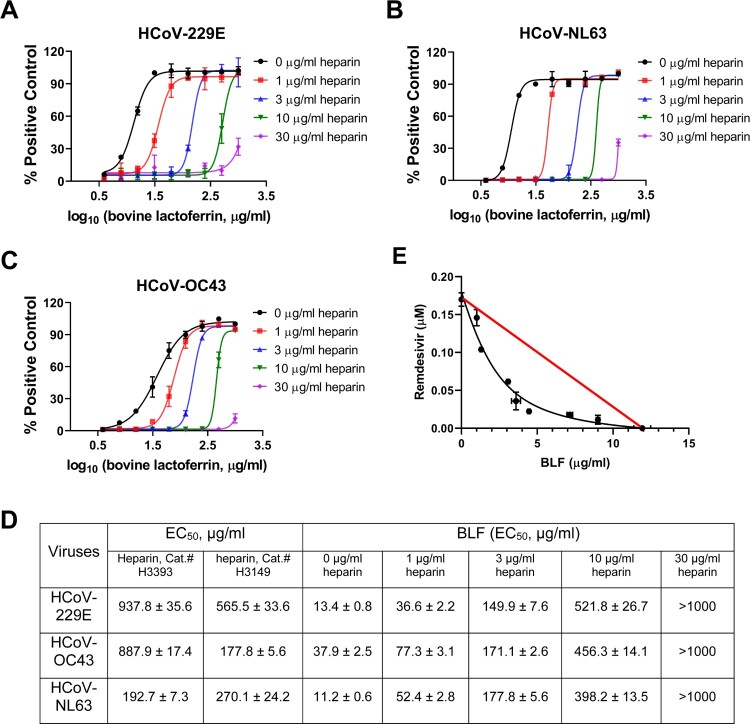
Antiviral potency of BLF in the absence or presence of different concentrations of heparin against HCoVs. Cells at near confluency were infected with (A) HCoV-229E; (B) HCoV-NL63; or (C) HCoV-OC43, different concentrations of BLF and indicated concentrations of heparin were mixed and immediately added into the cells for EC_50_ determination. EC_50_ curve fittings were obtained using log_10_ (concentration of inhibitors) vs. percentage of positive control (uninfected cells) with variable slopes in prism 8. (D) Antiviral EC_50_ values of BLF in the presence of different concentrations of heparin. (E) Combination therapy of remdesivir with BLF. Data are mean ± standard deviation of three replicates.

As illustrated above, BLF was shown to block viral attachment to the host cells and this inhibitory effect was neutralized by pre-mixing with heparin, therefore, it is expected that the cellular antiviral activity of BLF will decrease in the presence of heparin. To this end, the antiviral potency of BLF against HCoV-229E, HCoV-NL63 and HCoV-OC43 were determined in the absence or presence of different concentrations of heparin (Figure 7(A–C)). We first determined the antiviral activity of heparin from two different sources (Cat.# H3393 and Cat.# H3149) in CPE assay in cell cultures. Both heparins had EC_50_ values of a few hundred micromoles against all three HCoVs tested (Figure 7(D)), which is consistent with previous reported results [35]. To avoid the interference of antiviral activity from heparin, four concentrations of heparin (1, 3, 10 and 30 µg/ml), which are far below its minimum inhibitory concentration, were chosen to determine the effect of heparin on the antiviral potency of BLF in the CPE assay. Heparin decreased the antiviral activity of BLF against all three HCoVs tested in a dose-dependent manner (Figure 7(A–C)), with the EC_50_ values of BLF increased from 2 to >100-folds in the presence of heparin (Figure 7(D)).

### BLF has synergistic antiviral effect with remdesivir in cell culture

Combination therapy has been extensively explored for the treatment in oncology, parasitic, bacterial and viral infections [36–38], it has many advantages over monotherapies such as delayed development of drug resistance; synergistic efficacy on treatment; and fewer side effects due to the lower doses of drugs used. The combination treatment potential of BLF with remdesivir was explored using HCoV-OC43 antiviral CPE assay. Remdesivir is a SARS-CoV-2 polymerase inhibitor and is an FDA-approved antiviral. Combination indices (CIs) versus the EC_50_ values of compounds at different combination ratios were plotted as previously described [39]. The red line indicates additive effect; the right upper area above the red line indicates antagonism, while the left bottom area below the line indicates synergy [39]. In all combination scenarios, the CIs at all the combination ratios fell below the red line (Figure 7(E)), suggesting BLF displayed synergistic antiviral effect with remdesivir in the combination therapy.

### Modelling the binding between BLF and HSPGs

**Figure 8.**
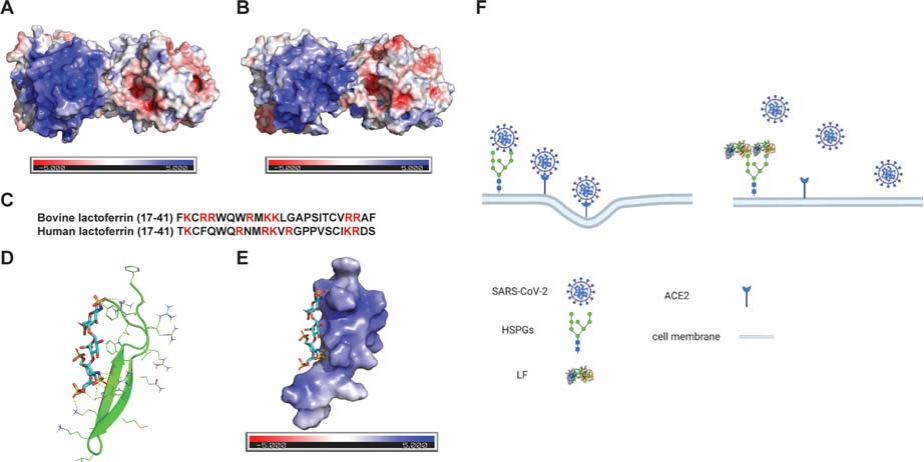
Structures of BLF, HLF, and the docking model of heparin dp4 with bovine lactoferricin. (A) Surface electrostatics of BLF (PDB: 1BLF). (B) Surface electrostatics of HLF (PDB: 1LFI). (C) Sequence alignment of the positively charged N-terminal domain of BLF and HLF. (D) Docking model of heparin dp4 with bovine lactoferricin (PDB: 1LFC). The heparin dp4 structure was from PDB 5E9C. (E) Surface view of the docking model of heparin dp4 with bovine lactoferricin. Docking was performed using AutoDock Vina and figures were generated using PyMOL. (F) Proposed antiviral mechanism of LF. The figure was created with BioRender.com.

LF is a single chain 80-kDa iron-binding glycoprotein that belongs to the transferrin family. It contains two symmetric N- and C-globular lobes. Multiple studies have shown that the heparin-binding site of BLF is located at the N-terminal domain of the N-lobe, which contains a cluster of positive charges spanning residues 17–41 [40]. This positively charged domain confers to the antiviral activity against adenovirus [41], papillomavirus [42], and echovirus 6 [43]. The trypsin digested N-terminal peptide lactoferricin alone is sufficient to prevent adenovirus, herpes simplex virus (HSV), and cytomegalovirus (CMV) infection [41,44,45]. It was found that the N-terminal domain of BLF is more negatively charged than HLF (Figure 8(A) vs. 8(B), and 8(C)), which might explain the more potent antiviral activity of BLF than HLF in inhibiting human papillomavirus, HSV, and HCMV [42,44,45]. Our data similarly showed that BLF is more potent than HLF in inhibiting common human coronaviruses including HCoV-OC43, HCoV-229E, and HCoV-NL63 (Figure 1(A) vs. 1(B)). To gain insights how BLF binds to HSPGs, we chose the bovine lactoferricin (PDB: 1LFC) [46] and dp4 (PDB: 5E9C) [47] as structural models of BLF and HSPGs, respectively. Docking model showed that the negatively charged sulfate in dp4 engages multiple electrostatic interactions with the positive charges from Arg25, Lys27, and Arg39 (Figure 8(D,E)).

Collectively, the proposed antiviral mechanism of action of LF against coronaviruses is shown in Figure 8(F). SARS-CoV-2 viral particles are recruited to cell surface by interaction with HSPGs, facilitating subsequent specific binding to ACE2 receptor and following internalization of the virion. LF binds to cell surface HSPGs, which blocks the interaction between SARS-CoV-2 and HSPGs and subsequent viral attachment to host cells.

## Discussion

Several studies have shown that HSPGs are co-receptors for the SARS-CoV-2 infection [48]. Due to their negative charges, HSPGs help to recruit SARS-CoV-2 to the cell surface by interacting with the viral spike protein, thereby increasing the local concentration of the viral spike protein for more effective subsequent binding with ACE2. A recent computational model suggested that a positively charged binding groove located at the viral spike protein RBD might be the putative binding site for the negatively charged HSPGs [23,24]. Binding study using surface plasma resonance assay showed that the monomer and trimer forms of SAS-CoV-2 spike glycoprotein bind to heparin with *K_d_* values of 4.0 × 10^−11^ and 7.3 × 10^−11^ M, respectively [24]. In addition to SARS-CoV-2, SARS-CoV similarly relies on HSPGs as an anchor for viral attachment [17]. Likewise, HCoV-NL63 is also known to utilize its membrane protein to bind to HSPGs and facilitate tethering of virions to the host cell surface [18,19,49]. A drug repurposing screening identified several compounds targeting HSPGs and HSPGs-dependent endocytosis pathways as potent entry inhibitors for SARS-CoV and SARS-CoV-2 [26]. Given the importance of HSPGs in coronavirus cell entry, it is plausible that compounds interfering with the binding between virus and HSPGs might inhibit viral replication. In this context, the endogenous natural protein LF becomes a prominent candidate due to its strong binding with HSPGs. Indeed, LF was previously shown to inhibit the SARS-CoV pseudovirus infection by blocking its interaction with HSPGs [17]. However, the antiviral activity of LF against infectious coronaviruses including SARS-CoV-2, HCoV-OC43, HCoV-NL63, and HCoV-229E has not been reported. Nevertheless, these results collectively suggest that HSPGs are a promising antiviral drug target for broad-spectrum antivirals against coronaviruses. In this study, we investigated the antiviral activity and mechanism of action of lactoferrins (BLF and HLF) against multiple coronaviruses. New findings and highlights of our study include: (1) BLF and HLF have broad-spectrum antiviral activity against infectious SARS-CoV-2, HCoV-OC43, HCoV-NL63, and HCoV-229E viruses in cell culture. It is noted that in parallel to our study, two preprints in biorxiv independently confirmed the antiviral activity of LF against SARS-CoV-2 [50,51]. (2) The inhibition of SARS-CoV-2 pseudovirus replication by BLF is not cell dependent, suggesting BLF might offer protection against SARS-CoV-2 infection in multiple tissues and organs. (3) The antiviral mechanism of action of BLF is through binding to host cell surface HSPGs (Figure 8(F)). Using SARS-CoV-2 pseudovirus and HCoV-OC43 as representative examples, we have shown that BLF inhibits viral attachment to the host cell and has no effect on viral entry and subsequent replication processes. The antiviral activity of BLF is diminished dose-dependently by exogenously added heparin. (4) BLF has synergistic antiviral effect with the FDA-approved SARS-CoV-2 antiviral remdesivir in cell culture. Collectively, this study provided compelling evidence to support the translational potential of LF as broad-spectrum antivirals for coronaviruses including SARS-CoV-2.

## Materials and methods

### Cell lines, viruses and reagents

Human RD, Vero E6 cell, Huh-7 cell, HEK293T cell expressing ACE2 (293T-ACE2), and HCT-8 cell lines were maintained in Dulbecco’s modified Eagle’s medium (DMEM); Caco-2 cell, Calu-3 cell and MRC-5 cell lines were maintained in Eagle's Minimum Essential Medium (EMEM). Both mediums were supplemented with 10% fetal bovine serum (FBS) and 1% penicillin–streptomycin antibiotics. Cells were kept at 37 °C incubator in a 5% CO_2_ atmosphere. The following reagent was obtained through BEI Resources, NIAID, NIH: Human Coronavirus, OC43, NR-52725 and was propagated on HCT-8 cell or RD cell; HCoV-229E was obtained from Dr. Bart Tarbet (Utah State University) and amplified on Huh-7 cell or MRC-5 cell; The following reagent was obtained through BEI Resources, NIAID, NIH: Human Coronavirus, NL63, NR-470 and propagated on Caco-2 cell or HEK293T cell expressing ACE2 cell (293T-ACE2).

BLF (Cat.# L9507), HLF (Cat.# L4894) and heparin sodium salt from porcine intestinal mucosa (Cat.# H3149 and Cat.# H3393) were purchased from Millipore Sigma (St. Louis, MO). LF were dissolved in sterile Nanopure water or PBS buffer with stock concentration of 10 mg/ml, heparin was dissolved in sterile Nanopure water with stock concentration of 50 mg/ml. Remdesivir was purchased from MedChemExpress and dissolved in DMSO before use.

### Antiviral assays

Antiviral activities of BLF or HLF against 229E, NL63 and OC43 were tested in CPE assays as previously described [52] with minor modifications. Briefly, cell cultures at near confluency in 96-well plates were infected with 100 µL of viruses at desired dilutions and incubated for 1 h. Unabsorbed virus was removed and different concentrations of LF (0, 3.9, 7.8, 15.6, 31.3, 62.5, 125, 250, 500, 1000 µg/ml) were added. Remdesivir was included as a positive control. The plates were incubated for another 3–5 days when a significant cytopathic effect was observed in the wells without compound (virus only). Cells were stained with 0.1 mg/ml neutral red for 2 h, and excess dye was rinsed from the cells with PBS. The absorbed dye was eluted from the cells with buffer containing 50% ethanol and 1% glacial acetic acid. Plates were read for optical density determination at 540 nm. Readings were normalized with uninfected controls. EC_50_ values were determined by plotting percent CPE versus log_10_ compound concentrations from best-fit dose response curves with variable slope in Prism 8. Toxicity of LF at each concentration was determined in uninfected cells in the same microplates by measuring neutral red dye uptake.

VYR assays were conducted as previously described [53] with minor modifications. Viruses were first replicated in the presence of different concentrations of BLF. Supernatant was harvested 2 dpi from each concentration of test compound and the viral yield was determined by plaque reduction assay. The EC_50_ values were calculated from best-fit dose response curves with variable slope in Prism 8.

Viral growth curves were obtained by replicating viruses in the presence or absence of BLF at MOI of 0.1. Viruses in the supernatant were collected at the indicated time-point post infection and viral titers were determined by plaque reduction assay. For all antiviral assays, LF was included during virus infection and post-infection stages.

### Time of addition

Drug time-of-addition experiment was performed as previously described [54,55]. Briefly, RD cells were seeded at 1 × 10^5^ cells/well in 12-well plate. 1000 µg/ml BLF was added at different time points of viral life cycle, as illustrated in Figure 2(B): −2 to −1 h (viral attachment), −1 to 0 h (viral entry), 0, 3, 6 to 14 h (post-viral entry). RD cells were infected with HCoV-OC43 at an MOI of 0.1 24 h after seeding. Viruses were harvested at 12 hpi. The viral titers were determined by plaque assay.

### Immunofluorescence imaging

HCoV-OC43 immunofluorescence staining was performed similarly as previously described [56] with minor modifications. For drug time-of-addition experiment using immunofluorescence staining, RD cells were infected with HCoV-OC43 at an MOI of 1. Viral infection started at the −2 h time-point and incubated at 4 °C for 1 h to allow virus attachment. At −1 h time-point, unbound virus was removed and cells were washed with ice-cold PBS buffer. Same volume of medium without virus was added into each well and cells were incubated at 33 °C for 1 h to allow virus entry. At 14 h post infection (hpi), cells were fixed with 4% formaldehyde for 10 min followed by permeabilization with 0.2% Triton X-100 for another 10 min. After blocking with 5% bovine serum, cells were sequentially stained with anti-Coronavirus antibody, HCoV-OC43 strain, clone 541-8F (Cat#: MAB9012, Millipore Sigma, Burlington, Massachusetts, USA) as primary antibody, and anti-mouse secondary antibody conjugated to Alexa-488 or Alexa-546 (Cat # A-11029, Cat # A-11030, Thermo Scientific, Waltham, Massachusetts, USA). Nuclei were stained with 300 nM DAPI (Cat#: D1306, Thermo Scientific, Waltham, Massachusetts, USA) after secondary antibody incubation.

### Pseudovirus assay

A pseudotype HIV-1-derived lentiviral particles bearing SARS-CoV-2 Spike and a lentiviral backbone plasmid encoding luciferase as reporter was produced in HEK293T cells engineered to express the SARS-CoV-2 receptor, ACE2 (ACE2/293T cells), as previously described [27]. The pseudovirus was then used to infect Vero E6 cells or Calu-3 cells or ACE2/293T cells in 96-well plates in the presence of H_2_O or serial concentrations of E-64d, Camostat Mesylate or BLF or HLF. 48 hpi, cells from each well were lysed using the Bright-Glo Luciferase Assay System (Cat#: E2610, Promega, Madison, WI, USA), and the cell lysates were transferred to 96-well Costar flat-bottom luminometer plates. The relative luciferase units (RLUs) in each well were detected using Cytation 5 Cell Imaging Multi-Mode Reader (BioTek, Winooski, VT, USA).

### Differential scanning fluorimetry (DSF)

The binding of heparin and BLF was monitored by DSF using a Thermal Fisher QuantStudio^™^ 5 Real-Time PCR System as previously described [57] with minor modifications. TSA plates were prepared by mixing BLF (final concentration of 100 μg/ml) with different concentrations (0.02–200 µg/ml) of heparin and incubated at 30 °C for 1 h. 1 × SYPRO orange (Thermal Fisher) were added and the fluorescence of the plates were taken under a temperature gradient ranging from 20 to 95 °C (incremental steps of 0.05 °C/s). The melting temperature (*T_m_*) was calculated as the mid-log of the transition phase from the native to the denatured protein using a Boltzmann model in Protein Thermal Shift Software v1.3. Δ*T_m_* was calculated by subtracting reference melting temperature of proteins in the presence of H_2_O from the *T_m_* in the presence of heparin. Curve fitting was performed using the Boltzmann sigmoidal equation in Prism (v8) software.

### Virus attachment assay

Viral attachment assay was performed as previously described [58]. RD cells or Vero E6 cells at 80–90% confluency were precooled at 4 °C for 30 min, followed by infection with HCoV-OC43 (MOI of 40) or HCoV-NL63 (MOI of 30). After 2 h incubation at 4 °C, unbound viruses were removed by washing the cells two times with ice-cold PBS. The infected cells with viruses attached on cell surface were harvested for quantification by real-time qPCR or fixed for visualization by immunofluorescence staining.

### RNA extraction and real-time PCR

RNA extraction and RT–PCR were performed as previously described [59]. Total RNA was extracted using TRIzol reagents (Thermo Fisher Scientific). About 2.0 μg of total RNA was used to synthesize the first strand cDNA of viral RNA and host RNA using SuperScript III reverse transcriptase (Thermo Fisher Scientific) and Random Hexamer primers. After digestion of genomic DNA with RQ1 RNase-free DNase (Promega), target gene was amplified on a Thermal Fisher QuantStudio^TM^ 5 Real-Time PCR System (Thermo Fisher Scientific) using FastStart Universal SYBR Green Master mix (carboxy-X-rhodamine; Roche) and following HCoV-NL63 N gene-specific primers (Forward: 5'-CTGTTACTTTGGCTTTAAAGAACTTAGG-3’; Reverse: 5'-CTCACTATCAAAGAATAACGCAGCCTG-3’) or HCoV-OC43 N gene-specific primers (Forward: 5'-CGATGAGGCTATTCCGACTAGGT-3’; Reverse: 5'- CCTTCCTGAGCCTTCAATATAGTAACC-3’). GAPDH was also amplified to serve as a control using human GAPDH-specific primers (GAPDH-F: 5′-ACACCCACTCCTCCACCTTTG-3′ and GAPDH-R: 5′-CACCACCCT GTTGCTGTAGCC-3′). The amplification conditions were: 95 °C for 10 min; 40 cycles of 15 s at 95 °C and 60 s at 60 °C. Melting curve analysis was performed to verify the specificity of each amplification. All experiments were repeated three times independently.

### Molecular modelling of the binding of LF to HS

Lactoferricin and dp4 were chosen as structural models for BLF and HSPGs, respectively. AutoDock Vina was used for modeling of binding of lactoferricin to dp4 [60]. Bovine lactoferricin (PDB: 1LFC) was set as the receptor and the docking grid box parameters were defined as the following: center_x = 32.636, center_y = 76.438, center_z = 46.275; size_x = 106, size_y = 120, size_z = 104; exhaustiveness = 40. The heparin dp4 structure was downloaded from PDB code 5E9C. The final docking poses were generated in PyMOL. The protein electrostatistics surface was generated using the APBS Electrostatistics model in PyMOL.

### Statistical analysis

All experiments were performed in duplicates or triplicates. Data are shown as mean ± S.D. Statistical analysis was performed using GraphPad Prism Software 8.0 (GraphPad Software Inc., La Jolla, CA, USA). Comparisons were performed using two-tailed Student’s *t*-test.

### Combination therapy

BLF was mixed with remdesivir at combination ratios of 8:1, 4:1, 2:1, 1:1, 1:2, 1:4, and 1:8, separately. The mixture of each compound with remdesivir at each combination ratio was serially diluted into 7 different concentrations and applied in HCoV-OC43 CPE assay to determine EC_50_ of BLF and remdesivir in the combination ratio. CIs plot was used to depict the EC_50_ values of each compound and remdesivir at different combination ratios. The red line indicates the additive effect, and above the red line indicates the antagonism, while below the red line indicates the synergy [39].
